# Immune Checkpoint Blockade for Metastatic Uveal Melanoma: Patterns of Response and Survival According to the Presence of Hepatic and Extrahepatic Metastasis

**DOI:** 10.3390/cancers13133359

**Published:** 2021-07-04

**Authors:** Elias A. T. Koch, Anne Petzold, Anja Wessely, Edgar Dippel, Anja Gesierich, Ralf Gutzmer, Jessica C. Hassel, Sebastian Haferkamp, Bettina Hohberger, Katharina C. Kähler, Harald Knorr, Nicole Kreuzberg, Ulrike Leiter, Carmen Loquai, Friedegund Meier, Markus Meissner, Peter Mohr, Claudia Pföhler, Farnaz Rahimi, Dirk Schadendorf, Beatrice Schell, Max Schlaak, Patrick Terheyden, Kai-Martin Thoms, Beatrice Schuler-Thurner, Selma Ugurel, Jens Ulrich, Jochen Utikal, Michael Weichenthal, Fabian Ziller, Carola Berking, Markus V. Heppt, on behalf of the German Dermatologic Cooperative Oncology Group (DeCOG, Committee Ocular Melanoma)

**Affiliations:** 1 Department of Dermatology, Universitätsklinikum Erlangen, Friedrich-Alexander-University Erlangen-Nürnberg (FAU), 91054 Erlangen, Germany; elias.koch@uk-erlangen.de (E.A.T.K.); Anne.Petzold@uk-erlangen.de (A.P.); Anja.Wessely@uk-erlangen.de (A.W.); Beatrice.Schuler-Thurner@uk-erlangen.de (B.S.-T.); Carola.Berking@uk-erlangen.de (C.B.); 2 Comprehensive Cancer Center Erlangen-European Metropolitan Area of Nuremberg (CCC ER-EMN), 91054 Erlangen, Germany; 3 Department of Dermatology, Ludwigshafen Medical Center, 67059 Ludwigshafen, Germany; dippele@klilu.de; 4 Department of Dermatology, University Hospital Würzburg, 97080 Würzburg, Germany; gesierich_a@ukw.de; 5 Skin Cancer Center Minden, Department of Dermatology, Mühlenkreiskliniken AöR, Ruhr University Bochum Campus Minden, 32423 Minden, Germany; Ralf.Gutzmer@ruhr-uni-bochum.de; 6 Skin Cancer Center, Department of Dermatology and National Center for Tumor Diseases (NCT), University Hospital Heidelberg, 69120 Heidelberg, Germany; Jessica.Hassel@med.uni-heidelberg.de; 7 Department of Dermatology, University Hospital Regensburg, 93053 Regensburg, Germany; sebastian.haferkamp@ukr.de; 8 Department of Ophthalmology, Universitätsklinikum Erlangen, Friedrich-Alexander-University Erlangen-Nürnberg (FAU), 91054 Erlangen, Germany; Bettina.Hohberger@uk-erlangen.de (B.H.); Harald.Knorr@uk-erlangen.de (H.K.); 9 Department of Dermatology, University Hospital Schleswig-Holstein, Campus Kiel, 24105 Kiel, Germany; kckaehler@yahoo.de (K.C.K.); mweichenthal@dermatology.uni-kiel.de (M.W.); 10 Department of Dermatology and Venereology, Skin Cancer Center at the Center of Integrated Oncology (CIO) Köln Bonn, University Hospital of Cologne, 50937 Cologne, Germany; nicole.kreuzberg@uk-koeln.de; 11 Department of Dermatology, Center for Dermatooncology, University Hospital Tübingen, 72056 Tübingen, Germany; Ulrike.Leiter@med.uni-tuebingen.de; 12 Department of Dermatology, University Medical Center Mainz, 55131 Mainz, Germany; carmen.loquai@unimedizin-mainz.de; 13 Skin Cancer Center at the University Cancer Centre Dresden and National Center for Tumor Diseases & Department of Dermatology, University Hospital Carl Gustav Carus, 01307 Dresden, Germany; Friedegund.Meier@uniklinikum-dresden.de; 14 Department of Dermatology, Venereology and Allergology, Goethe University, 60590 Frankfurt am Main, Germany; markus.meissner@kgu.de; 15 Department of Dermatology, Elbeklinikum, 21614 Buxtehude, Germany; peter.mohr@elbekliniken.de; 16 Department of Dermatology, Saarland University Medical School, 66421 Homburg/Saar, Germany; claudia.pfoehler@uks.eu; 17 Department of Dermatology and Allergy, Munich University Hospital (LMU), 81377 Munich, Germany; Farnaz.Rahimi@med.uni-muenchen.de; 18 Department of Dermatology, University Hospital Essen, University Duisburg-Essen, 45147 Essen, Germany; Dirk.Schadendorf@uk-essen.de (D.S.); Selma.Ugurel@uk-essen.de (S.U.); 19 German Cancer Consortium, Partner Site Essen, 45147 Essen, Germany; 20 Department of Dermatology, SRH Wald-Klinikum Gera, 07548 Gera, Germany; Beatrice.Schell@srh.de; 21 Department of Dermatology, Venerology and Allergology, Charité—Universitätsmedizin Berlin, Corporate Member of Freie Universität Berlin, Humboldt-Universität zu Berlin and Berlin Institute of Health, 10117 Berlin, Germany; max.schlaak@charite.de; 22 Department of Dermatology, University of Lübeck, 23562 Lübeck, Germany; patrick.terheyden@uksh.de; 23 Department of Dermatology, University Medical Center Goettingen, 37075 Goettingen, Germany; kai.thoms@med.uni-goettingen.de; 24 Department of Dermatology, Harzklinikum Dorothea Christiane Erxleben, 06484 Quedlinburg, Germany; jens.ulrich@harzklinikum.com; 25 Skin Cancer Unit, German Cancer Research Center (DKFZ), 68167 Heidelberg, Germany; Jochen.Utikal@umm.de; 26 Department of Dermatology, Venereology and Allergology, University Medical Center Mannheim, Ruprecht-Karl University of Heidelberg, 68167 Mannheim, Germany; 27 Department of Dermatology, DRK Krankenhaus Rabenstein, 09117 Chemnitz, Germany; Ziller.Fabian@drk-khs.de

**Keywords:** uveal melanoma, immune checkpoint blockade, PD-1, CTLA-4, liver metastasis, treatment resistance

## Abstract

**Simple Summary:**

This retrospective multicenter study examines the influence of hepatic and extrahepatic metastases on the response of immune checkpoint blockade (ICB) in patients with metastatic uveal melanoma. A better response to dual ICB was observed in the presence of extrahepatic metastases in two recently published phase II trials. Therefore, we investigated two cohorts with and without extrahepatic metastasis and have assembled a population of 178 patients treated with ICB. The survival of this large cohort of patients with advanced UM was more favorable than that reported in previous benchmark studies. Patients with both hepatic and extrahepatic metastasis showed more favorable survival and higher response to dual ICB than those with hepatic metastasis only.

**Abstract:**

Background: Since there is no standardized and effective treatment for advanced uveal melanoma (UM), the prognosis is dismal once metastases develop. Due to the availability of immune checkpoint blockade (ICB) in the real-world setting, the prognosis of metastatic UM has improved. However, it is unclear how the presence of hepatic and extrahepatic metastasis impacts the response and survival after ICB. Methods: A total of 178 patients with metastatic UM treated with ICB were included in this analysis. Patients were recruited from German skin cancer centers and the German national skin cancer registry (ADOReg). To investigate the impact of hepatic metastasis, two cohorts were compared: patients with liver metastasis only (cohort A, *n* = 55) versus those with both liver and extra-hepatic metastasis (cohort B, *n* = 123). Data were analyzed in both cohorts for response to treatment, progression-free survival (PFS), and overall survival (OS). The survival and progression probabilities were calculated with the Kaplan–Meier method. Log-rank tests, χ^2^ tests, and t-tests were performed to detect significant differences between both cohorts. Results: The median OS of the overall population was 16 months (95% CI 13.4–23.7) and the median PFS, 2.8 months (95% CI 2.5–3.0). The median OS was longer in cohort B than in cohort A (18.2 vs. 6.1 months; *p* = 0.071). The best objective response rate to dual ICB was 13.8% and to anti-PD-1 monotherapy 8.9% in the entire population. Patients with liver metastases only had a lower response to dual ICB, yet without significance (cohort A 8.7% vs. cohort B 16.7%; *p* = 0.45). Adverse events (AE) occurred in 41.6%. Severe AE were observed in 26.3% and evenly distributed between both cohorts. Conclusion: The survival of this large cohort of patients with advanced UM was more favorable than reported in previous benchmark studies. Patients with both hepatic and extrahepatic metastasis showed more favorable survival and higher response to dual ICB than those with hepatic metastasis only.

## 1. Introduction

At least 40–50% of patients with uveal melanoma (UM), depending on the genetic background of the primary tumor, develop metastases, which spread predominantly to the liver [1]. Since there is no standardized and effective treatment for advanced UM, the prognosis remains poor once metastasis develops [2]. A meta-analysis of studies published between 1980 and 2017 including 2494 patients calculated a median OS across all treatment modalities of 1.07 years [3]. However, the population of this meta-analysis was treated mainly in the time before immune checkpoint blockade (ICB) was available and some patients show a more favorable disease course with longer OS. In analogy to the use in cutaneous melanoma (CM), ICB includes the antibodies anti-CTLA-4 (ipilimumab), anti-PD-1 (nivolumab, pembrolizumab), and the combination of anti-PD-1 with anti-CTLA-4 (dual ICB). Two recent phase II trials investigated the value of dual ICB in metastatic UM. Piulats et al. reported that the OS in patients with exclusive liver metastases was shorter than that in patients with metastases in locations other than the liver and those with both liver and other metastases [4]. Pelster et al. achieved a response in 6 patients of whom 5 had both liver and extrahepatic metastasis [5]. These results imply that the presence of liver metastasis only may represent an unfavorable prognostic factor for response to ICB. To further dissect the role of hepatic metastasis on the response of ICB we aimed to compare two cohorts of patients with and without extrahepatic metastasis from UM in a real-world setting.

## 2. Materials and Methods

### 2.1. Patient Population and Study Design

We performed a retrospective multi-center explorative analysis. Patients with metastatic UM receiving any ICB (ipilimumab, nivolumab, pembrolizumab, dual ICB) were eligible. A total of 178 patients were included and divided into two cohorts. Cohort A comprised patients with liver metastases only (*n* = 55, cohort A) while cohort B included those with several metastatic sites (i.e., hepatic and extra-hepatic, *n* = 123, cohort B). Patients without liver metastases were excluded. Clinical data and the treatment outcomes of interest were extracted from the original patient records from 15 German skin cancer centers (Erlangen *n* = 55, Tübingen *n* = 19, München *n* = 18, Mainz *n* = 7, Mannheim *n* = 5, Frankfurt *n* = 4, Kiel *n* = 4, Dresden *n* = 3, Köln *n* = 3, Göttingen *n* = 2, Heidelberg *n* = 2, Homburg *n* = 2, Ludwigshafen *n* = 2, Lübeck *n* = 2, Würzburg *n* = 2), as well as from the prospective multicentric skin cancer registry ADOReg of the German Dermatologic Cooperative Oncology Group (DeCOG) (*n* = 48). The ADOReg collects data for high-quality real-world evidence studies; all ADOReg patient IDs included in this study were checked for duplicates. The data were collected and merged into a central database before analysis. This study was approved by the institutional review board of the medical faculty of the Munich University Hospital (approval number 413-16 UE) and was conducted following the principles of the Helsinki Declaration in its current version.

### 2.2. Data Collection and Treatment Outcomes

The recorded clinical data at baseline comprised demographics with sex, age, number of organ systems affected by metastasis, and date of death or last documented patient contact. At the date of ICB start, the Eastern Cooperative Oncology Group (ECOG) performance status and serum lactate dehydrogenase (LDH) levels were collected from patient charts and analyzed for their prognostic value. Regarding the treatment, we recorded the number and type of therapies, ICB start date, date of progression during ICB, the best response to ICB (based on the RECIST criteria version 1.1), adverse event assessment, and grading based on the CTCAE criteria (version 5) and if the patients received radiation or liver-directed treatment. We summarized any metastases besides liver, bone, pulmonary, CNS, lymph node, connective tissue, and skin metastases as a category “other metastases.”

OS was calculated as the time from ICB start until melanoma-specific or treatment-related death. PFS was determined as the time from treatment start until disease progression confirmed by radiologic imaging or clinically evident (if radiologic imaging lacking because of decline in clinical condition). Complete (CR) and partial (PR) response were summarized as objective response rate (ORR). Time-to-event analyses were calculated where death or disease progression was considered as events. If neither occurred or if patients were lost to follow-up, the date of the last documented presentation was used as a censored observation.

### 2.3. Statistical Analyses

The survival and progression probabilities were calculated with the Kaplan–Meier method. Log-rank tests were performed to compare the survival and progression probabilities of the two cohorts. Furthermore, χ^2^ tests and t-tests were conducted (i) to test the comparability of the two cohorts, i.e., concerning possible different baseline characteristics, and (ii) to compare the response to ICB of both cohorts. In all cases, two-tailed *p*-values were calculated and considered significant with values *p* < 0.05. Patients with missing values for a given variable were excluded. No imputation of missing data was performed. All analyses were carried out with the software R (https://www.r-project.org/ (accessed on 1 March 2021) using the packages “survival” and “survminer”.

## 3. Results

### 3.1. Baseline Patient Characteristics

A total of 178 patients with metastatic UM were included. Eighty-two percent (*n* = 146) were naïve to systemic treatment and received ICB as the first-line therapy; 49.4% of patients had an ECOG status of 0 (*n* = 88). The serum LDH was elevated in 50% of cases (*n* = 89) at baseline. Both parameters were evenly distributed among both cohorts (54.5% vs. 44.7% and 50.9% vs. 49.6%, respectively). The patients had predominantly metastases to the liver (100%), lung (46.1%), bone (26.4%), lymph node (23%), CNS (14%), skin (13.5%), connective tissue (4.5%) and in 28.7% “other metastases.” Other baseline characteristics are listed in detail in Table 1.

### 3.2. Response Rates to ICB

Dual ICB was applied in 109 patients (61.2%; cohort A 69.1% vs. cohort B 57.7%), while 15 patients received ipilimumab monotherapy (8.4%; cohort A 0% vs. cohort B 12.2%). PD-1 inhibitors were given as monotherapy in 53 patients (29.8%; cohort A 30.9% vs. cohort B 29.3%). The best ORR to dual ICB was 13.8% and to anti-PD-1 monotherapy 8.9% in the entire population (27 patients were not evaluable for radiologic response). No patients achieved a CR, while 17 patients had a PR. Patients with liver metastases only (cohort A) showed a numerically worse response to dual ICB, yet without significance (cohort A 8.7% vs. cohort B 16.7%; *p* = 0.45). In contrast, the ORR to single PD-1 inhibition was numerically higher in cohort A, albeit not significantly (cohort A 14.3% vs. cohort B 6.5%; *p* = 0.77). Details of the patterns of response to ICB are summarized in Table 2. ICB was given on average for 2.0 months (95% CI 0–13.0) and 2.1 months (95% CI 0–24.4) in cohorts A and B, respectively (*p* = 0.14).

### 3.3. Survival Data

**Figure 1:**
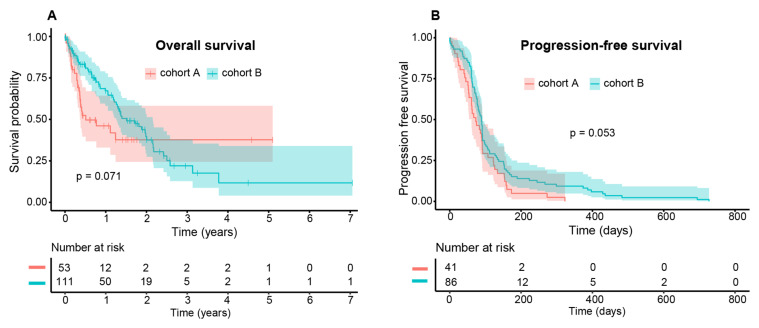
Kaplan–Meier estimates of the patient population for (**A**) OS and (**B**) PFS to ICB comparing cohort A (red) vs. B (turquoise). Although there was no significant difference in OS and PFS (*p* = 0.071 and *p* = 0.053, resp.), the median survival differed considerably (cohort A 6.1 months (95%-CI: 4.4-NA) vs. cohort B 18.2 months (95%-CI: 15.1–25.9)). In contrast, the median PFS only differed slightly (cohort A 2.4 months (95%-CI: 2.0–3.0) vs. cohort B 2.9 months (95%-CI 2.5–3.0)).

**Figure 2:**
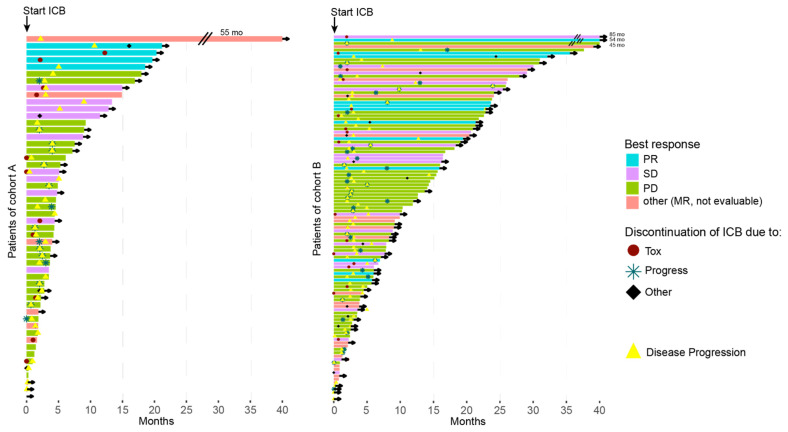
Swimmer plots for cohort A (**left**) and B (**right**) demonstrating the OS for each patient. The color shows the best response to ICB, the symbols depict the reason for treatment discontinuation and the yellow triangle shows the time of tumor progression. If a patient was censored, an arrow is drawn.

The entire cohort showed a median OS of 16 months (95% CI 13.4–23.7) and a median PFS of 2.8 months (95% CI 2.5–3.0) to any ICB. There was a statistical trend in OS (*p* = 0.071) and PFS (*p* = 0.053) for both cohorts. The median values differed conspicuously for OS (cohort A 6.1 months (95% CI 4.4-not available) vs. cohort B 18.2 months (95% CI 15.1–25.9)). In contrast, the median PFS was similar in both cohorts (cohort A 2.4 months (95% CI 2.0–3.0) vs. cohort B 2.9 (95% CI 2.5–3.0)) (Figure 1). The survival was also more favorable in cohort B evident in a swimmer’s plot comparing both cohorts (Figure 2).

### 3.4. Adverse Events (AE)

A total of 133 AE were reported in 74 (41.6%) patients. Of all events, 72 AE (54.1%) were graded as severe (grade 3–5), with no difference between both cohorts (*p* = 0.93). These 72 events were observed in 47 patients (26.3%; cohort A 14 patients (29.1%) vs. cohort B 31 patients (25.2%); *p* = 0.72). The treatment was discontinued in 36 cases due to unacceptable toxicity. One death occurred in cohort A during treatment but was most likely due to disease progression. The most common events were colitis (*n* = 30), hepatitis (*n* = 19), thyroiditis (*n* = 13), hypophysitis (*n* = 8), pancreatitis (*n* = 6), myalgia with myositis (*n* = 5), and cutaneous toxicity (*n* = 6). No significant differences were detected between both cohorts (Table 3; Supplementary Materials Table S1).

## 4. Discussion

Here, we present to our knowledge the hitherto largest published cohort of patients with metastatic UM who were treated with ICB. We detected a median OS of 16 months (95% CI 13.4–23.7) and a median PFS of 2.8 months (95% CI 2.5–3.0) to any ICB. There was considerably better OS and PFS in patients with both hepatic and extra-hepatic metastatic sites (cohort B), albeit without reaching statistical significance (*p* = 0.071 and *p* = 0.053, respectively). The median OS of 16 months is higher compared to the previous benchmark survival studies done before the ICB era [3,6]. We conclude that the prognosis of patients with metastatic UM has improved due to the availability and more frequent use of ICB [7,8]. The ORR to dual ICB of 13.8% remains low compared to the ORR in cutaneous and mucosal melanoma. There were no significant differences between the ORR values of the cohorts and only a slight tendency toward higher ORR to dual ICB in patients with both hepatic and extrahepatic metastasis. The ORR is in line with another previously published retrospective study reporting 11.6% to dual ICB [9] as well as to our previous report of 15.6% [10]. Two recently published prospective trials of the combination of ipilimumab with nivolumab in patients with metastatic UM showed results that slightly deviate [4,5]. Piulats et al. enrolled only treatment-naïve patients and reported a lower OS (12.7 vs. 19.1 months), PFS (3 vs. 5.5 months), and ORR (11.5% vs. 18%) compared to Pelster et al., who enrolled patients with any number of prior treatments. Interestingly, Piulats et al. enrolled also more patients with ECOG 0 (84.6% vs. 71%) and fewer patients with elevated LDH (32% vs. 43%), resulting in an unfavorable ORR in a prognostically favorable population [11]. Furthermore, Piulats et al. reported a median number of two liver metastases (range 1–25) and a median size of the biggest liver metastases of 25 mm (range 10–90 mm); 78.8% of the patients had liver metastases and 57.7% presented with extrahepatic disease. The number of patients with exclusive liver metastases was not presented [4]. In comparison, Pelster et al. reported that 49% of patients presented with hepatic and extrahepatic metastases, 31% with liver metastases only, and 20% with extrahepatic metastasis only [5]. The comparison of both cohorts of this population revealed that patients with exclusive liver metastases had a poorer OS and PFS after ICB. In contrast, patients with both hepatic and extrahepatic metastases had higher ORR to dual ICB (8.7% vs. 16.7%) although this difference was not significant. Thus, it remains unclear if the survival benefits observed in cohort B with both hepatic and extrahepatic metastatic sites are specifically due to ICB treatment or if this cohort is prognostically favorable regardless of the treatment with ICB.

In CM, liver metastases are the least responsive metastatic site to dual ICB with a median of 3% tumor regression compared to other metastatic sites with a median of 77% [12]. Mechanistically, macrophages induce apoptosis of CD8+ T cells in the immunosuppressive microenvironment of the liver through fas-ligand binding. This results in an elimination of CD8+ T cells possibly explaining ineffective tumor control and poor response to immunotherapy [13,14,15]. The comparison of CM to UM liver metastases has demonstrated that there is no difference in the extent of immune infiltration, but UM showed a higher ratio of exhausted CD8+ T cells to cytotoxic T cells, total CD8+ T cells, and Th1 cells. In addition, a higher and more frequent PD-L1 expression on CM liver metastases, as well as higher TMB was found compared to those from UM [16]. This may also contribute to the worse treatment response in UM as a low TMB is associated with poor response to ICB while higher PD-L1 expression is predictive for PD-1 inhibitor response [17,18]. It was further shown that PD-1 and PD-L1 expression is generally very low in UM [19]. However, PD-L1 expression was not considered in this study.

AE occurred in 20.8% of patients with anti-PD-1 monotherapy, in 55% with dual ICB, and severe AE in 7.5% with anti-PD-1 monotherapy and 31.2% of patients with dual ICB with no difference between cohorts A and B (Table 3). The rate of severe AE is in line but on the lower range of previously published studies where immune-related grade 3/4 toxicities in dual ICB occurred in about 30–60% of patients [4,5,9,10].

The major limitation of this study is its retrospective design. In particular, the quantification of the exact extent of metastases and tumor burden in the liver was difficult based on chart reviews. According to our data, we could only assess whether liver metastases were present or not. Furthermore, the quality and completeness of the data, in particular the reporting of AE, is highly dependent on the participating cancer centers. Thus, we cannot exclude that AE were underreported in this study.

## 5. Conclusions

Our data of 178 patients with advanced UM treated with ICB demonstrates an improved OS compared to studies conducted before the ICB era. Counterintuitively, patients with several metastatic sites seem to have a favorable prognosis compared to patients with hepatic metastasis only. If this phenomenon is related to ICB response warrants further investigation. Nevertheless, our results imply that exclusive hepatic metastases are a major unfavorable prognostic factor.

## Figures and Tables

**Table 1:** Characteristics of the study population. Abbreviations: NA = not available, ICB = immune checkpoint blockade.

		Total	Cohort A	Cohort B	A vs. B
Sex	Women	89 (50.0%)	25 (45.5%)	64 (52.0%)	*p* = 0.52
	Men	89 (50.0%)	30 (54.5%)	59 (48.0%)	
Age	Median in years (range)	65.6 (17.7–87.6)	63.4 (32.4–87.6)	65.8 (17.7–85.4)	*p* = 0.79
LDH	Not elevated	42 (23.6%)	16 (29.1%)	26 (21.1%)	*p* = 0.34
	Elevated	89 (50.0%)	28 (50.9%)	61 (49.6%)	
	NA	47 (26.4%)	11 (20.0%)	36 (29.3%)	
ECOG	ECOG 0	88 (49.4%)	30 (54.5%)	58 (44.7%)	*p* = 0.44
	ECOG 1	20 (11.2%)	3 (5.5%)	17 (13.8%	
	ECOG 2	4 (2.2%)	1 (1.8%)	3 (2.4%)	
	ECOG 3	2 (1.1%)	1 (1.8%)	1 (0.8%)	
	ECOG 4	0 (0.0%)	0 (0.0%)	0 (0.0%)	
	ECOG 5	0 (0.0%)	0 (0.0%)	0 (0.0%)	
	NA	64 (36.0%)	20 (36.4%)	44 (35.8%)	
Number of affected organ systems	Median (range)	2 (1–7)	1	3 (2–7)	*p* < 0.001
Affected organ systems	Liver	178 (100%)	55 (100%)	123 (100%)	
	Pulmonary	82 (46.1%)	0 (0.0%)	82 (66.7%)	
	Bone	47 (26.4%)	0 (0.0%)	47 (38.2%)	
	CNS	25 (14.0%)	0 (0.0%)	25 (20.3%)	
	Lymph node	41 (23.0%)	0 (0.0%)	41 (33.3%)	
	Connective tissue	8 (4.5%)	0 (0.0%)	8 (6.5%)	
	Skin	24 (13.5%)	0 (0.0%)	24 (19.5%)	
	Disseminated	10 (5.6%)	0 (0.0%)	10 (8.1%)	
	Other	51 (28.7%)	0 (0.0%)	51 (41.5%)	
ICB as first-line therapy		146 (82.0%)	49 (89.1%)	97 (78.9%)	*p* = 0.15
Other therapies	Gemcitabine & Treosulfan	34 (19.1%)	4 (7.2%)	30 (24.3%)	*p* = 0.013
	Nivolumab	38 (21.3%)	10 (18.1%)	28 (22.7%)	*p* = 0.623
	Pembrolizumab	25 (14%)	6 (10.9%)	19 (15.4%)	*p* = 0.568
	Sorafenib	18 (10.1%)	1 (1.8%)	17 (13.8%)	*p* = 0.029
	DC vaccine	14 (7.8%)	2 (3.6%)	12 (9.7%)	*p* = 0.26
	Dacarbazine	9 (5%)	0 (0%)	9 (7.3%)	*p* = 0.091
	Trametinib	10 (5.6%)	2 (3.6%)	8 (6.5%)	*p* = 0.678
	Fotemustine	9 (5%)	2 (3.6%)	7 (5.6%)	*p* = 0.835
ICB substance	Any	177 (99.4%)	55 (100%)	122 (99.2%)	*p* = 1
	anti-PD-1 (pembrolizumab, nivolumab)	53 (29.8%)	17 (30.9%)	36 (29.3%)	*p* = 0.97
	anti-CTLA-4 (ipilimumab)	15 (8.4%)	0 (0.0%)	15 (12.2%)	*p* = 0.016
	Dual	109 (61.2%)	38 (69.1%)	71 (57.7%)	*p* = 0.20
	NA	1 (0.6%)	0 (0.0%)	1 (0.8%)	

**Table 2:** Response rates to ICB according to ICB substance. Abbreviations: CR = complete response, PR = partial response, SD = stable disease, PD = progressive disease, ORR = objective response rate, DCR = disease control rate.

ICB—Any Type	Total	Cohort A	Cohort B	Test (Cohorts A vs. B)
CR	0/150 (0.0%)	0/48 (0.0%)	0/102 (0.0%)	
PR	17/150 (11.3%)	5/48 (10.4%)	12/102 (11.8%)	*p* = 1
SD	36/150 (24.0%)	11/48 (22.9%)	25/102 (24.5%)	*p* = 0.99
PD	91/150 (60.7%)	30/48 (62.5%)	61/102 (59.8%)	*p* = 0.89
ORR	17/150 (11.3%)	5/48 (10.4%)	12/102 (11.8%)	*p* = 1
DCR	53/150 (35.3%)	16/48 (33.3%)	37/102 (36.3%)	*p* = 0.87
**anti-PD-1**	**Total**	**Cohort A**	**Cohort B**	**Test (Cohorts A vs. B)**
CR	0/45 (0.0%)	0/14 (0.0%)	0/31 (0.0%)	
PR	4/45 (8.9%)	2/14 (14.3%)	2/31 (6.5%)	*p* = 0.77
SD	9/45 (20%)	2/14 (14.3%)	7/31 (22.6%)	*p* = 0.81
PD	30/45 (66.7%)	10/14 (71.4%)	20/31 (64.5%)	*p* = 0.91
ORR	4/45 (8.9%)	2/14 (14.3%)	2/31 (6.5%)	*p* = 0.77
DCR	13/45 (28.9%)	4/14 (28.6%)	9/31 (29.0%)	*p* = 1
**Dual ICB**	**Total**	**Cohort A**	**Cohort B**	**Test (Cohorts A vs. B)**
CR	0/94 (0.0%)	0/34 (0.0%)	0/60 (0.0%)	
PR	13/94 (13.8%)	3/34 (8.7%)	10/60 (16.7%)	*p* = 0.45
SD	25/94 (26.6%)	9/34 (26.5%)	16/60 (26.7%)	*p* = 1
PD	52/94 (55.3%)	20/34 (58.8%)	32/60 (53.3%)	*p* = 0.77
ORR	13/94 (13.8%)	3/34 (8.7%)	10/60 (16.7%)	*p* = 0.45
DCR	38/94 (40.4%)	12/34 (35.3%)	26/60 (43.3%)	*p* = 0.59
**anti-CTLA-4**	**Total**	**Cohort A**	**Cohort B**	**Test (cohorts A vs. B)**
CR	0/11 (0.0%)	0/0	0/11 (0.0%)	Not possible
PR	0/11 (0.0%)	0/0	0/11 (0.0%)	
SD	2/11 (18.2%)	0/0	2/11 (18.2%)	
PD	9/11 (81.8%)	0/0	9/11 (81.8%)	
ORR	0/11 (0.0%)	0/0	0/11 (0.0%)	
DCR	2/11 (18.2%)	0/0	2/11 (18.2%)	

**Table 3:** Occurrence of adverse events. Abbreviations: AE = adverse events.

	Total	Cohort A	Cohort B	Test (Cohorts A vs. B)
Number of AE	133	43	90	
Number of severe AE	72 (54.1%)	24 (55.8%)	48 (53.3%)	*p* = 0.93
Number of patients with AE	74 (41.6%)	26 (47.3%)	48 (39.0%)	*p* = 0.39
Number of patients with severe AE	47 (26.3%)	16 (29.1%)	31 (25.2%)	*p* = 0.72
		**anti-PD1**	**Dual ICB**	**Test (Cohorts A vs. B)**
Number of AE		25	103	
Number of severe AE (grade 3 + 4)		6 (24.0%)	60 (58.3%)	*p* = 0.0044
Number of patients with AE		11 (20.8%)	60 (55.0%)	*p* < 0.001
Number of patients with severe AE (grade 3 + 4)		4 (7.5%)	34 (31.2%)	*p* = 0.0017

## Data Availability

Data are contained within the article or Supplementary Materials.

## Supplementary Materials

The following are available online at https://www.mdpi.com/article/10.3390/cancers13133359/s1, Table S1: Baseline characteristics between single PD1 and dual ICB therapy.
