# Supplementary material for: Immune Checkpoint Blockade for Metastatic Uveal Melanoma: Patterns of Response and Survival According to the Presence of Hepatic and Extrahepatic Metastasis
Source: Cancers (Basel). 2021 Jul 4;13(13):3359. doi: 10.3390/cancers13133359 (PMC8268645; doi:10.3390/cancers13133359)
Supplement: Supplementary file 1 [file cancers-13-03359-s001.zip › cancers-1271724-supplementary.pdf]

Supplementary Materials:

Immune Checkpoint Blockade for Metastatic Uveal Melanoma: Patterns of Response and Survival According to the Presence of Hepatic and Extrahepatic Metastasis

Elias A. T. Koch, Anne Petzold, Anja Wessely, Edgar Dippel, Anja Gesierich, Ralf Gutzmer, Jessica C. Hassel, Sebastian Haferkamp, Bettina Hohberger, Katharina C. Kähler, Harald Knorr, Nicole Kreuzberg, Ulrike Leiter, Carmen Loquai, Friedegund Meier, Markus Meissner, Peter Mohr, Claudia Pföhler, Farnaz Rahimi, Dirk Schadendorf, Beatrice Schell, Max Schlaak, Patrick Terheyden, Kai-Martin Thoms, Beatrice Schuler-Thurner, Selma Ugurel, Jens Ulrich, Jochen Utikal, Michael Weichenthal, Fabian Ziller, Carola Berking, Markus V. Heppt and on behalf of the German Dermatologic Cooperative Oncology Group (DeCOG, Committee Ocular Melanoma)

Table S1. Baseline characteristics between single PD1 and dual ICB therapy.

1.	2.	3. Single PD1 (N=53)	4. Combi (N=109)	5. Test
6. Sex	7. Women	8. 28 (52.8%)	9. 54 (49.5%)	10. p=0.82
	11. Men	12. 25 (47.2%)	13. 55 (50.5%)	
14. Age	15. Median (range)	16. 65.4 (31.4-83.6)	17. 65.6 (17.7-87.6)	18. p=0.99
19. LDH	20. not elevated	21. 11 (20.8%)	22. 30 (27.5%)	23. p=0.46
	24. Elevated	25. 27 (50.9%)	26. 55 (50.5%)	
	27. NA	28. 15 (28.3%)	29. 24 (22.0%)	
30. ECOG	31. ECOG 0	32. 24 (45.3%)	33. 60 (55.0%)	34. p=0.32
	35. ECOG 1	36. 2 (3.8%)	37. 14 (12.8%)	
	38. ECOG 2	39. 3 (5.7%)	40. 1 (0.9%)	
	41. ECOG 3	42. 1 (1.9%)	43. 1 (0.9%)	
	44. ECOG 4	45. 0 (0.0%)	46. 0 (0.0%)	
	47. ECOG 5	48. 0 (0.0%)	49. 0 (0.0%)	
	50. NA	51. 23 (43.4%)	52. 33 (30.3%)	
53. Number of affected organ systems	54. Median (range)	55. 2 (1-7)	56. 2 (1-6)	57. p=0.34
58. Affected organ systems	59. Liver	60. 53 (100%)	61. 109 (100%)	62. p=0.44
	63. Pulmonary	64. 26 (49.1%)	65. 45 (41.3%)	

	66. Bone	67. 13 (24.5%)	68. 26 (23.9%)	
	69. CNS	70. 7 (13.2%)	71. 12 (11.0%)	
	72. Lymph node	73. 16 (30.2%)	74. 17 (15.6%)	
	75. Connective tissue	76. 4 (7.5%)	77. 4 (3.7%)	
	78. Skin	79. 12 (22.6%)	80. 8 (7.3%)	
	81. Disseminated	82. 3 (7.5%)	83. 5 (4.6%)	
	84. Other	85. 16 (30.2%)	86. 27 (24.8%)	
	87. NA	88. 0 (0.0%)	89. 0 (0.0%)	
90. CPI as first-line therapy	91.	92. 42 (79.2%)	93. 97 (89.0%)	94. p=0.15
95. Number of pre-therapies	96. Median (range)	97. 0 (0-3)	98. 0 (0-1)	99. p=0.064
